# Perceived usefulness of technology and multiple salient outcomes: the improbable case of oil and gas workers

**DOI:** 10.1016/j.heliyon.2022.e09322

**Published:** 2022-04-26

**Authors:** Precious Bolanle Bolodeoku, Ebeguki Igbinoba, Paul Odunayo Salau, Charles Kelechi Chukwudi, Sandra Efeomo Idia

**Affiliations:** Department of Business Administration, Covenant University, Nigeria

**Keywords:** Perceived usefulness, Technology, Salient outcomes, Oil and gas sector

## Abstract

This research aimed at assessing the effect perceived usefulness of technology on multiple salient outcomes in Nigeria. Specifically determining how employee performance affects the perceived usefulness of technology in the oil and gas sector. The study used a descriptive research design. The target population of the study was 495 employees of selected oil and gas firms in Nigeria who have been recognized to have adopted strategies to communicate the usefulness of adopted technology in their business operations. This study used the purposive sampling technique to collect data from the employees of the selected firm. The questionnaire was used as the main data collection instrument, on a population of 495 and a total of 460 was collected back for the study analysis. A descriptive research design was adopted to establish trends related to the objectives of this study. Specifically, this research used a quantitative method (questionnaire) for the collection of data while structural equation modeling was used to analyze the data collected. The result showed that the perceived usefulness of technology has a significant effect on salient outcomes on workers in the oil and gas. Hence, the result shows that the perceived usefulness of technology contributes more to employees' satisfaction, organizational support, and employees' productivity while employees' commitment had the least. This study recommended that organizations in the oil and gas industry have to increase their efforts using strategies to improve the use of adopted technologies to promote employees’ commitment. However, organizations are to maintain strategies of perceived usefulness of technology on organizational support.

## 1 Introduction

The oil and gas industry has been considered the engine for the growth and development of the economy both locally and globally (Cocchi and Mazzeo, 2021). This is becoming increasingly vital for the oil and gas industry to set organizational standards in other to improve salient outcomes of employees. To this effect and bridging a digital gap, the main objective of this study is to determine the effect of perceived usefulness of technology on employee's performance in the oil and gas sector. However, organizations have experienced labor shortages due to recent transitions in the global economy as a result of the Covid-19 pandemic (Mordor, 2021). The Covid-19 pandemic has affected different global economic sectors, leading to a high turnover rate and fewer organizational activities. Thus, organizations are demanding Increased salient outcomes, especially employee performance, particularly from oil firms (Deliotte, 2020). However, to achieve the desired performance of employees, organizations' standards have to be set and communicated to employees; to assess performance and analyze the contrast between actual and standard measures on performance (Ogueyungbo et al., 2019). The performance of employees can be motivated through factors such as employee commitment, satisfaction, and productivity.

Emeka (2021), posit that poor performance of employees can be a result of lack of recognition, unfair compensation, insufficient role speculation, and lack of empowerment which leads to a decrease in employees' commitment, organizational support, employees satisfaction, employees productivity, organizational citizenship behavior, and leadership role (Yuvaraj and Nadheya, 2018). Employees' performance, high or low, can sometimes be caused by non-monetary issues in various organizations, especially in the oil and gas industry. As a result, managers and leaders in various organizations focused on adopting effective strategies to facilitate the long-term impact of employees' performance (Anwar, 2018). Therefore, technological innovation has been said to enhance the performance of employees thus leading to overall organizational performance. The need for technological innovation in the oil and gas sector goes beyond increasing production but also, ensuring that workers’ safety is a priority (Coccia, 2020).

According to Davis (1989), technological innovation can be further explained using perceived ease of use, perceived usefulness of technology found under the Technology Acceptance model. However, in organizations, every individual has a perception of how useful and effortless it is to adopt technological innovations (Baaziz, 2021). When it comes to adapting to technology, individuals have to go through psychological adoption, technological perception gain and have an understanding of the importance of behavioral control (Baron and Kenny, 1986). Furthermore, the oil and gas industry's technological advances have consistently tuned and fine-tuned stages from the scene of exploration to the distribution of petroleum products and consumer usage (Total energies, 2021). Specifically, developing countries such as Canada, the United States, China has introduced technologies such as robotics, artificial intelligence, virtual reality, etc., as this has invaded the country to the point where employees' performance is affected both in the public and private sector both positively and negatively. According to Lonadek (2020) innovations such as robotics, artificial intelligence is rather new or foreign to some employees and a lack of required training, especially in a sector like oil and gas, would lead to an increase in the accident rate.

The oil and gas industry has come as far as using virtual reality, artificial intelligence, robotics, advanced seismic techniques in horizontal drilling and hydraulic fracturing, floating liquified gas, floating production storage, and many more innovations when carrying out different specific or general functions (Salihu, 2018).

Perceived usefulness is an individual's perception of how technologies or a particular technology are set to improve the individuals' tasks or roles in terms of efficiency and effectiveness (Robert, However, individuals are different and react to change differently. Not every individual is comfortable moving from how things have been done to a new method. Nevertheless, when organizations do not properly communicate or demonstrate how the adopted technology improves effectiveness and efficiency in line with their job roles, there is bound to be a reduction in employee performance (Abbas et al., 2021). As a result of this, organizations have to aid the process of technology adoption by effectively communicating the benefits and usefulness of adopting new processes or products to enhance employee performance (Naveena, 2019). Nonetheless, relatively few relevant studies have been performed concerning this topic (Abbas et al., 2021). To bridge a digital gap, the main objective of this study is to determine the effect of the perceived usefulness of technology on employee performance in the oil and gas sector. However, before the above objective, the following null hypothesis was developed for this research.

H0 = Perceived usefulness has no significant effect on employees’ performance.

## 2 Literature review

### 2.1 Perceived usefulness of technology

Malik and Annuar (2021) defined perceived usefulness as the potential user's subjective likelihood, which gives a probability that the technology used will improve the individual or team's performance from an organizational perspective. The operators' personal opinion of whether employing a given technology would improve performance reflects perceived usefulness (Davis et al., 1989).

The level at which the individual believes that the technology used may become a sole factor toward attaining their learning goals is the perceived utility of a technology. Perceived usefulness is a concept that shows how impacting individuals' behavior happens to be a specific factor of sustained usage on numerous occasions (Xia et al., 2019). Extrinsic and intrinsic motivation are the proper constructs that motivate the use of technology by people. The extrinsic motivation comes off as a type of enthusiasm that stems from the belief that the user may use technology to boost certain outcomes received from specific activities. According to Denny et al. (2021), the perceived usefulness variable should be measured or assessed using five different size indicators: advanced productivity, beneficial for the individual, effectiveness, faster transactions, and effectiveness of an activity. Employees’ beliefs about adopting mobile marketing products and services are strongly influenced by perceived relevance to their functions or tasks, especially when these products are affordable and present in the market (Mohammed, 2018). As a result, organizations must develop strategies that would communicate how useful a product or process is to their target market. Nonetheless, research has shown that when employees find are convinced that particular technology would enhance their performance, they are more likely to adopt it (Azman et al., 2020).

### 2.2 Salient outcomes (employees’ performance)

Employees' performance is critical to achieving organizational goals and objectives; employees' output impacts the organization's performance, which can be positive or negative. Organizations have lobbied for changes that have a beneficial impact on employees' performance due to the inevitability of change. However, while arguing for change, management must guarantee that issues that affect employees' performance should be evaluated (Umar and Udeme, 2021).

According to Shadare and Olaniyan (2021), various factors, ranging from changes in leadership, organizational structure, and technology adopted, have significant effects on employees' performance. Finding a technique to get the most out of an organization's employees' performance and production is one of the main objectives (Emeka, 2021). Performance measures a company's capability to achieve its goal and an individual's ability to apply their information and skills effectively and efficiently. According to Akilo and Olaosebikan (2021), employee performance is a critical activity that determines the goals and strategies for accomplishing organizational objectives and the level of output achievement.

Performance management is a critical component of organizational and technical efficiency (Nwobu et al., 2021). Businesses must find elements that boost employees’ performance to get the most out of them. Individuals' pay performance is entirely dependent on the policies, innovations, structures, tactics, and management of the organization in question regarding their pay package, incentives, bonuses, yearly increments, career growth, and other perks and benefits (Shathees et al., 2020). Globalization, economic upheaval, volatile commercial environments, continuously changing consumer expectations, and advanced technology all pose challenges to management in the oil and gas industry.

## 3 Theoretical framework

### 3.1 Technology acceptance model

Technology acceptance model understands the critical aspects that influence an innovation's acceptability (Venkatesh and Davis, 2000). Davis (1989) presents the theory of reasoned action (TRA) in such a way that it is determined on understanding and predicting customer adoption of information technology (Davis, 1989). Technology adoption is simply an individual's acceptance of particular information technology and information system (Davis, 1989). Significant aspects of the model of Technology Acceptance entail two sole constructs, perceived usefulness—which illustrates the importance and subjective capacities of persons to use computer base applications in a way that the individual attains utility at a maximum point to accomplish their work or role effectively. Considerably, perceived ease of use is linked to how an individual experiences easiness and is competent to use computer-based programs with fairly little effort. Moreover, the most prominent and extensively used theory for understanding an individual's embrace of information technology is the technology acceptance model (Amir and Radnejad, 2019). To comprehend user acceptability in information systems, the Technology acceptance model determines user behavior and acknowledges the role of perceived ease of use (PEOU) and perceived usefulness (PU) (Venkatesh and Davis, 2000). The technology acceptance model has been criticized for not properly representing the nature of consumer acceptance, even though it has been used in significant research (Moon and Kim, 2001).

## 4 Methodology

The study employed the use of a descriptive research design to help to achieve a reasonable representation of the various sub-groups on a population of 495. The selection of the organization involved was based on their performance. The stratified random method was used to enhance the efficacy of the samples. However, the population is categorized into various strata (selection of staff across departments), and to eliminate bias selection, employees were randomly selected across departments. The survey was performed using a questionnaire.

The questionnaire distributed is broken down into sections A and B. Respondents ‘demographic profile is found in section A (age, sex, contract type), in contrast, Section B features the independent variable (technological innovation) and dependent variable (employees’ performance). Adopted were the Five Likert-scale questions in the research which range from strongly agree to strongly disagree (strongly agree-5, agree-4, undecided-3, disagree-2, strongly disagree-1). In this research, a structured questionnaire served as a research instrument, which enhanced the documentation of statistically significant results from the procedure of data analysis (Zikmund et al., 2010).

The data for the research was collected using structural copies of a questionnaire administered to staff of a selected oil and gas firm. The data collected back was a total of 460 employees, of which Structural equation model and Statistical Package for Social Science version 25 were used to collate and analyze the data. Smart Partial Least Square (SEM-PLS, version 3) software was used for the analysis because this tool can be used for theory testing in the early stages (Hoyle, 2011; Hair et al., 2013). However, the research instruments were coined out of previous studies such as Fernando and Adriana (2016), Hsien-Ta et al. (2003), Mohand (2020), Kuruppu et al. (2021).

This research was given ethical approval by the Covenant University Business Management Research Ethics Committee. However, before the researcher got consent from the participants/key respondents, the researcher ensured that the participants or key respondents to this research were well informed about the purpose as well as the background of the study, and these employees were kept informed with the whole data collection process. Therefore, respondents were given the choice or option of being anonymous while their responses were kept confidential.

## 5 Results

To evaluate the hypothesis formulated in this research, a Structured Equation Modeling (SEM) was used, but first, the result from the demographic characteristics of employees and frequency distribution of independent and dependent variables are explained in Table 1 and Table 2. Employees of selected oil and gas firm were asked to respond to a structured questionnaire. Data were collected between March–August 2021. A total of 460 responses were gotten back with a case of 35 data missing. More concretely, the collected data. All the validity concerns i.e., construct, convergent and discriminant validity are taken care of.

**Table 1:** Demographic characteristics of staff.

Demographic variables		Frequency	Percentage
Gender	Male	109	94.8
	Female	6	5.2
Marital Status	Single	40	34.8
	Married	70	60.9
	Divorced	5	4.3
Age	21–30	43	37.4
	31–40	28	24.3
	41–50	30	26.1
	51 – above	14	12.2
Highest Level Qualification	OND/NCE	7	6.1
	BSC/HND	34	29.6
	M.sc/MBA	44	38.3
	PhD	3	2.6
	Others	27	23.5
Work Duration	less than one year	19	16.5
	1–5years	34	29.6
	6–10years	37	32.2
	11–15years	17	14.8
	16 years- above	8	7.0
Staff Status	Permanent	73	63.5
	Contract	42	36.5
Departments	Electrical	13	11.3
	Mechanical	18	15.7
	Drilling	42	36.5
	Maintenance crew	34	29.6
	Operations	8	7.0

Source: Field Survey, 2021

**Table 2:** Factor loading for perceived usefulness of technology and employees' performance (employees' satisfaction, employees' commitment, employees’ productivity, and organizational support).

	Factor loading	Error variance	Composite reliability	AVE	Cronbach alpha	No. of indicators
Indicators	> 0.6	< 0.5	≥0.8	≥0.5	≥0.7	
**Perceived usefulness of technology**			**0.825**	**0.751**	**0.751**	**2**
B2	0.874	0.126				
B3	0.860	0.14				
**Employees' commitment**			**0.906**	**0.651**	**0.794**	**2**
F1	0.911	0.089				
F3	0.687	0.313				
**Employees' satisfaction**			**0.740**	**0.603**	**0.712**	**2**
E2	0.556	0.444				
E3	0.947	0.053				
**Organizational support**			**0.886**	**0.946**	**0.896**	**2**
G1	0.976	0.024				
G2	0.919	0.081				
**Employees' productivity**			**0.592**	**0.807**	**0.754**	**3**
H1	0.751	0.249				
H2	0.568	0.432				
H3	0.943	0.057				

### 5.1 Demographic characteristics of the respondents

Table 1 shows the demographic characteristics of the employees who work offshore in selected oil and gas firm. This study used descriptive statistics on the categorization of responses on demographic features of respondents.

Table 1 highlights the demographic characteristics of the respondents. The first section displays the gender of the respondents who participated in this study, 109 participants out of the total respondents are male with 94.8% while 6 participants out of the total respondents are females with 5.2%, although there is a large gap between both genders, it shows the organization takes into consideration gender diversity. The second section shows the marital status of the respondents who participated in this study, 40 participants were single with 34.8%, 70 were married with 60.9%, while 5 participants were divorced with a percentage of 4.3.

The third section displays the age range of respondents who participated in this study, 43 participants out of the total respondents are between the ages of 21–30 years old with 37.4%, 28 respondents who filled this questionnaire are between the ages of 31–40 years old with 24.3%, while 30 participants out of the total respondents are of the ages of 41–50 years of age with 26.1% and 14 participants out of the total respondents are 51 years old and above with 12.2%. The fourth section shows the Highest Educational Qualification of the respondents, 7 of the respondents are OND/NCE holders with 6.1%, 34 of the respondents are B.SC/HND holders with 29.6%, 44 of the respondents are M.Sc./MBA holders with 38.3%, while 3 of the respondents who filled the questionnaire for this study are Ph.D. holders with 2.6%, and 27 participants had other qualifications with 23.5%.

The fifth section shows the number of years in which the respondents have spent in their current organization, 19 of the respondents have spent less than one year with 16.5%, 34 of the respondents have spent between 1 to 5 years in the organization with 29.6%, 37 of the respondents have spent 6–10 years in the organization with 32.2%, while 17 (14.2%) participants have spent 11–15 years and 8 (7.0%) participants years and above. The sixth section shows the staff status, 73 (63.5%) happen to be permanent staff while 42(036.5%). Lastly, in the seventh section, predominant respondents came from the drilling department at 36.5%. Furthermore, the frequency distribution was arrived at with the use of SPSS. This implies that most of the respondents were well educated and experienced and their responses can be relied upon.

## 6 Test of hypothesis, data interpretation, and findings

**H0** = **Perceived usefulness of technology has no significant effect on Salient outcomes- employees’ performance**.

**Figure 1:**
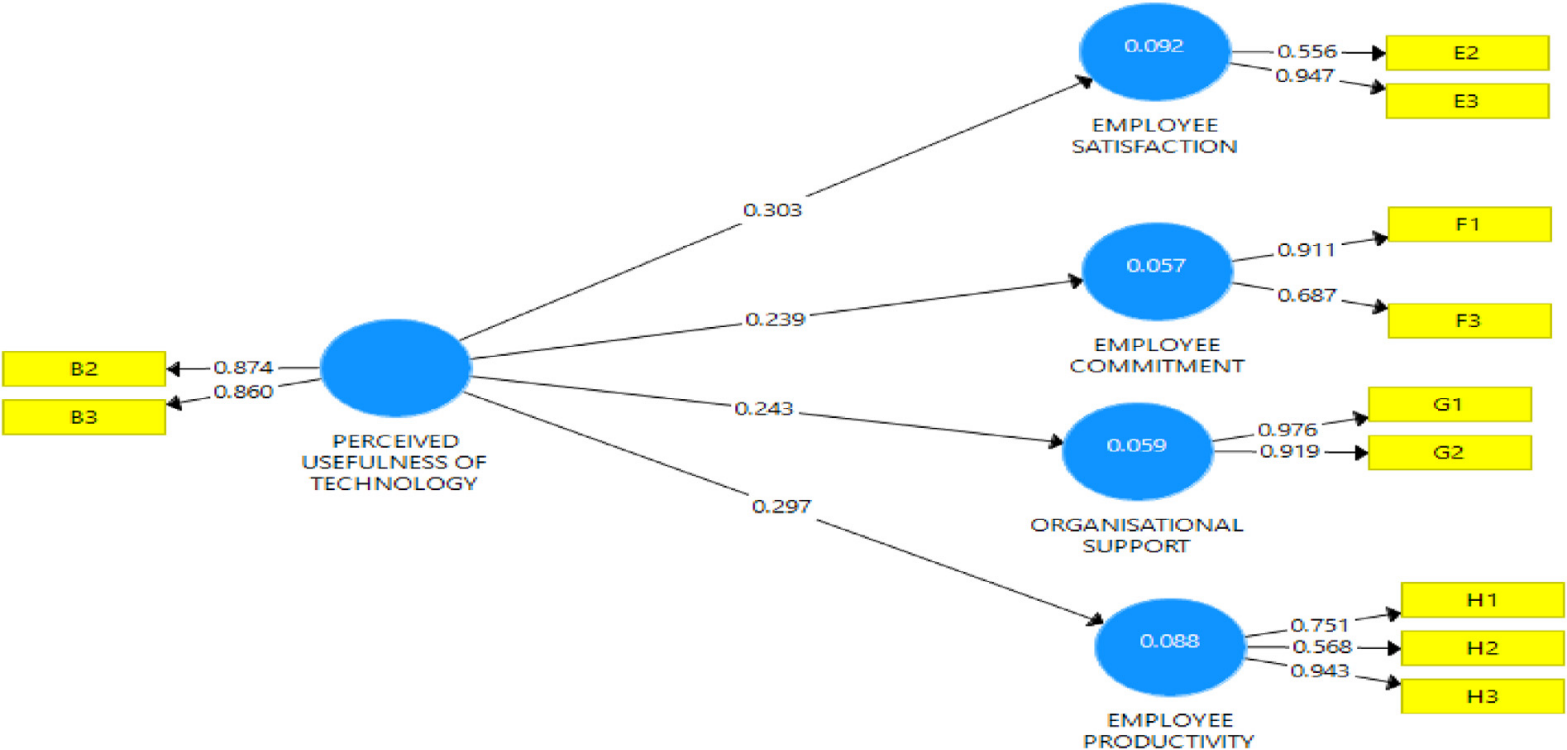
Predictive relevance (Path coefficient) of perceived usefulness of technology on employees' performance (employees' satisfaction, employees' commitment, employees’ productivity, and organizational support).

The research variables were measured using a structured questionnaire with a five Likert scale. The evaluation of Perceived usefulness of technology which is the latent variable was measured with three (3) items, while employees' performance was measured with twelve (9) items as shown in Table 3. The items adapted for measuring evaluation of employee performance, include employees' satisfaction, employees' commitment, employees' productivity, and organizational support. The factor loading depicted in Table 2 for all items on perceived usefulness of technology and employees’ performance was above the minimum threshold of 0.60 as suggested by (Fornell and Larcker, 1981; Newkirk and Lederer, 2006). Although items lower than the 0.500 value were removed and the results are presented in Figure 1 and Table 2. The instrument is adjudged reliable and valid since the fundamental requirement for the degree of fitness was satisfactorily met.

**Table 3:** Discriminant validity for salient outcomes.

	PUT	ES	EC	OS	EP
Perceived Usefulness of Technology (PUT)	**0.870**				
Employee Satisfaction (ES)	0.736	**0.782**			
Employee Commitment (EC)	0.601	0.700	**0.825**		
Organisational Supports (OS)	0.634	0.656	0.593	**0.764**	
Employee productivity (EP)	0.598	0.719	0.621	0.724	**0.819**

**Notes**: Values on the diagonal (bolded) are square root of the AVE while the off-diagonals are correlation.

The construct composite reliability must be equal to or greater than 0.80. However, the construct average variance extracted estimate (AVE) must be above the minimum threshold of 0.50. Finally, the Cronbach Alpha must be equal to or above 0.70 for the instruments to be reliable.

From the above Table, it can be shown that all constructs from perceived usefulness of technology and employees' performance have values higher than 0.60, which means that they have composite internal consistency and Cronbach Alpha reliability respectively. Under this hypothesis, the perceived usefulness of technology was examined employees' performance. The findings under Figure 1 also depict that the perceived usefulness of technology has a 9.2% effect on employees' satisfaction, 5.7% effect on employees' commitment, 5.9% effect on organizational support, and 8.8 % effect on employees’ productivity.

### 6.1 Discriminant validity

To support the path coefficient analysis, the convergent (i.e., discriminant) validity was assessed using Fornell and Larcker (1981) by comparing the square root of each AVE in the diagonal with the correlation coefficients (off-diagonal) for each construct in the relevant rows and columns. The most extensively used method for this is the Fornell and Larcker criterion (Hair et al., 2022). An assessment of the discriminant validity is shown in Table 3.

As presented in Table 3 the discriminant validity demonstrates that all the bold values are higher compared to the correlations. This means that the measures used to assess each dimension have a significant positive intercorrelation, and there are no collinearity issues among the latent components (multicollinearity). Thus, the discriminant validity is acceptable as it meets the Fornell–Larcker criterion.

### 6.2 The path coefficients (β) and T- statistics estimation

The bootstrapping for perceived usefulness of technology and employees' performance (i.e., employees' satisfaction, employees' commitment, organizational support, and employees' productivity) was presented in Figure 1 and Table 4. This hypothesis predicted that perceived usefulness of technology includes the adoption of multiple tools(B1), use of tools on oil field (B2), digital platform development (B3), significantly influence employees' performance (employees' satisfaction, employees' commitment, employees’ productivity, and organizational support) in the oil and gas sector in Nigeria as displayed in the table.

**Table 4:** Path coefficients for perceived usefulness of technology and employees' performance (i.e., employees' satisfaction, employees' commitment, organizational support, and employees’ productivity.

Variables and Cross Leading			Path co-efficient (O)	Std. Dev (STDEV)	T-statistics (O/STDEV)	P-values
Perceived usefulness of technology	→	Employees' commitment	0.239	0.116	2.066	0.039
Perceived usefulness of technology	→	Employees' satisfaction	0.303	0.125	2.420	0.016
Perceived usefulness of technology	→	Organizational support	0.243	0.095	2.565	0.011
Perceived usefulness of technology	→	Employees' productivity	0.297	0.127	2.339	0.020
				**R-Square (R**^**2**^**)**	**R-Square (R2) Adjusted**	
Perceived usefulness of technology	→	Organizational support		0.059	0.051	
Perceived usefulness of technology	→	Employees' productivity		0.088	0.080	
Perceived usefulness of technology	→	Employees' commitment		0.057	0.049	
Perceived usefulness of technology	→	Employees' satisfaction		0.092	0.084	

The path coefficient (R_2_) through PLS Algorithm model, and the PLS bootstrapping model with β, T values with a threshold of >1.96, and P values with a threshold of <0.005 as shown under Table 4, indicates the significant level of perceived usefulness of technology on employees’ performance.

**Figure 2:**
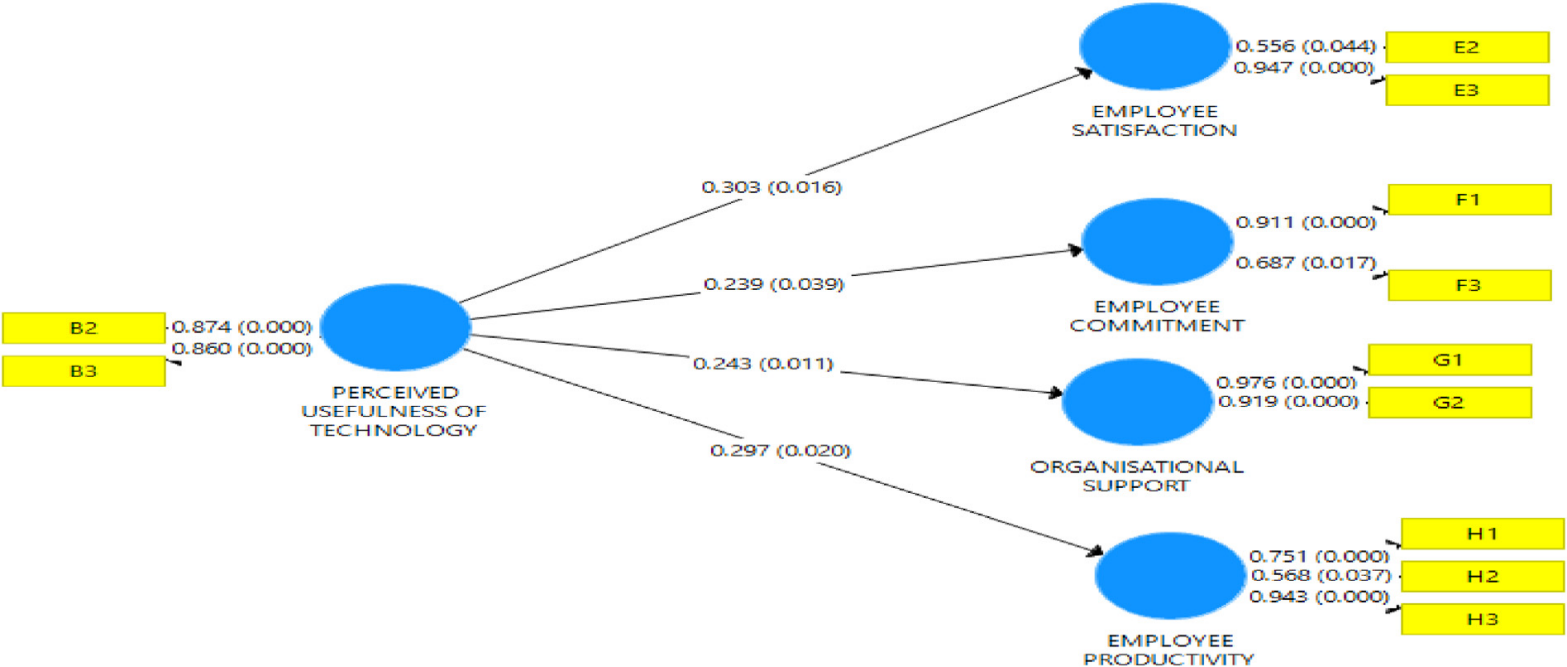
Path Co-efficient and P-values for perceived usefulness of technology and Employees' performance (i.e., employees' satisfaction, employees' commitment, employees’ productivity, and organizational support) in the oil and gas sector in Nigeria.

Figure 2 also indicated that there is a predictive power of the relationship between variables. The result established that holding the variables constant, the unit change in perceived usefulness of technology will lead to the increase in employees' commitment 23.9%, employees' satisfaction by 33.3%, organizational support by 24.3%, employees’ productivity by 29.7%.

The P-value of all constructs indicated in Figure 2 and Table 4 shows a significant influence in the analysis ≤0.05. The model indicated a statistically significant path coefficient between the variables and also show an increase in a unit of the exogenous variables which leads to an increase in the endogenous variables. Perceived usefulness of technology and employees' commitment (i.e., b = 0.239, p < 0.05, Tval = 2.066) perceived usefulness of technology on employees' satisfaction (i.e. b = 0.303, p < 0.5, Tval = 2.420), perceived usefulness of technology and employees' productivity (i.e. b = 0.297, p < 0.05, Tval = 2.339), perceived usefulness of technology and organizational support (i.e. b = 0.243, p < 0.5, Tval = 2.565). Hence, the result shows that the perceived usefulness of technology contributes more to employees' satisfaction, organizational support, and employees' productivity while employees’ commitment had the least. All the path coefficients were of practical importance since the significance level is below .05.

The R square result revealed the degree to which the variance in employee performance in the oil and gas industry is explained by the perceived usefulness of technology. According to Cohen (1988) in the social and behavioural sciences, R2 > 0.02 is classified as a modest effect, R2 > 0.13 as a medium effect, and R2 > 0.26 as a large effect.

However, the variance explained by the model was 59% for organizational support, 88% for employee productivity, 57% for employee commitment, 92% for employee satisfaction. In this research the least degree of R-square is 0.0057, which is expressed as 57%, meaning that out of all the variables, employees' commitment as a sub variable under employees’ performance, explains the perceived usefulness of technology the least. While employee satisfaction is explained by the perceived usefulness of technology at 92% (0.092) as the highest degree.

## 7 Discussion of findings

**H0: Perceived usefulness of technology has no significant effect on employees’ performance**.

Using the structural equation model, the study aimed at assessing the effect perceived usefulness of technology on multiple salient outcomes in Nigeria. The technology acceptance model was used as a basis to analyze the effect perceived usefulness of technology on multiple salient outcomes. The four endogenous variables had different interpretations in the aspect of being significant, the degree of variance to the exogenous variable, the relationship, and the effect of the endogenous variable on the endogenous variable.

The path coefficient indicated that the perceived usefulness of technology directly and significantly influences employees' performance in the analysis at <0.05, therefore the null hypothesis was rejected. The result also showed that the perceived usefulness of technology has a significant effect on employees’ performance in the selected oil and gas firm.

By implication, the findings suggest that when employees find the organization's technology valuable, to a little extent employees are prone to be committed to their jobs and as well the organization. However, the research depicted that when employees have a greater understanding of the value of technology adopted by the organization, it would lead to employees being satisfied with the organization's development to a large extent.

Moreover, the most prominent and extensively used theory for understanding an individual's embrace of information technology is the technology acceptance model. When employees are committed to their workplace, they will absorb all the information as a learning process, including the technology adopted and the system implemented by the company (Davis, 1989). Considerably, in this research, the perceived usefulness of technology does not improve employee commitment to a large extent, especially in the oil and gas firms in Nigeria. This research has therefore created a gap for future researchers, to discover other factors that aid employee commitment to a great extent and in general, employee performance in different sectors.

In line with the findings of perceived usefulness of technology by Panari et al. (2021), the belief in improved performance with new technology tools was the foundation for perceived usefulness. All of this means that, in order for technology to be tagged as useful, and interaction with it to be properly explained to employees, the technology's utility and interaction must be adequately articulated. Employees should be motivated to use new technologies with one of the aims being to improve performance.

The findings agreed with the research of Malik and Annuar (2021), Tala and Malak (2021), Okolocha (2021), who supported the notion that perceived usefulness is a potential user's subjective likelihood, which gives a probability that the technology used will improve the individual or team's performance from an organizational perspective. The operators' personal opinion of whether employing a given technology would improve performance reflects perceived usefulness.

## 8 Managerial implications and conclusion

The primary objective of this study is to assess the effect perceived usefulness of technology on multiple salient outcomes in the Nigerian oil and gas sector. It is evident that the use of technology influences employee performance. Thus, the study provides more understanding on the effect of perceived usefulness of technology on employee performance, taking into consideration variables like employee satisfaction, organizational support, employee productivity, and employee commitment. The importance of technology in the development of the nation's economy and the creation of competitive advantage for organizations cannot be emphasized enough.

By implication, the perceived usefulness of technology has a significant effect on multiple salient outcomes within organizations. Therefore, managers must pay extra attention to factors that elevate employee performance, in the light of employee satisfaction, organizational support, employee productivity, and employee commitment. Organizations in the oil and gas industry have to increase their efforts using strategies of perceived usefulness of technology control to promote employees' commitment, as failure to do this based on research leads to a high rate of turnover, reduction in profit, and so on. However, organizations are to maintain strategies of perceived usefulness of technology on employees' satisfaction and improve their strategies of perceived usefulness of technology on employees' commitment which would serve as an advantage to increase employees’ efficiency.

Further, it can be inferred from the findings that the effect of perceived usefulness of technology cannot be fully utilized, understood, or even serve as a positive factor if the required employee training and development is not addressed properly to fill up the gaps and this has been implemented by developing countries such as Canada, Germany, and the USA.

The adverse performance of employees experienced in the Nigerian oil and gas sector will be history if employees are well trained, educated, qualified, and confident about the technology and how it is considered useful to their different roles while saving time and delivering high results. This study has been able to add to the existing body of literature on the effect of perceived usefulness of technology on salient outcomes- employees’ performance in the oil and gas sector.

This paper offers practical implications and concludes that the deficiencies witnessed in Nigeria's oil and gas sector are attributed to issues such as lack of employee training, bureaucratic administrative machinery, policy reversals, the insufficient capacity of the majority of workers, certificate forgery to get promotion and gain entry, age falsification to remain in service beyond the stipulated age, primordial considerations like ethnicity, etc. By implications, this study suggests that the perceived usefulness of technology should be tailored towards ensuring operations in this sector will facilitate employees' performance in terms of employee satisfaction, organizational support, employee productivity and employee commitment.

## 9 Recommendations

Based on the empirical findings and theoretical findings above, the research objectives of carrying out this research has been reached and these recommendations have been made concerning this field as a study:

i. asides the involvement of technological innovation, compensation strategies, working conditions, and environment should be continuously improved to enhance the employee productivity and organizational profitability in the oil and gas industry;
ii. organizations in the oil and gas industry have to increase their efforts using strategies to improve the use of adopted technologies to promote employees' commitment; and
iii. as technology keeps advancing and the oil and gas industry continuously receives new methods and equipment for operation, it is imperative to offer practical training to enhance the adoption of technology of employees. Nevertheless, training with the use of virtual reality, artificial intelligence, game apps would help in practicing with heavy-duty equipment used in this sector such as hydraulic fracturing, seismic technology, gas frac, etc. as this would also help reduce contact with employees in the organization.

## Declarations

### Author contribution statement

Precious Bolanle Bolodeoku: Conceived and designed the experiments; Analyzed and interpreted the data.

Odunayo Paul Salau: Performed the experiments; Analyzed and interpreted the data.

Charles Kelechi Chukwudi: Performed the experiments.

Ebeguki Igbinoba: Contributed reagents, materials, analysis tools or data.

Sandra Efeomo Idia: Contributed reagents, materials, analysis tools or data; Wrote the paper.

### Funding statement

This work was supported by Covenant University Centre for Research, Innovation and Discovery.

### Data availability statement

The authors are unable or have chosen not to specify which data has been used.

### Declaration of interests statement

The authors declare no conflict of interest.

### Additional information

No additional information is available for this paper.

## Appendix A Supplementary data

questioniare PERCEIVED USEFULNESS OF TECHNOLOGY questioniare PERCEIVED USEFULNESS OF TECHNOLOGY
