# Supplementary material for: Perceived usefulness of technology and multiple salient outcomes: the improbable case of oil and gas workers
Source: Heliyon. 2022 Apr 26;8(4):e09322. doi: 10.1016/j.heliyon.2022.e09322 (PMC9062673; doi:10.1016/j.heliyon.2022.e09322)
Supplement: questioniare PERCEIVED USEFULNESS OF TECHNOLOGY [file mmc1.docx]

**PERCEIVED USEFULNESS OF TECHNOLOGY AND MULTIPLE SALIENT OUTCOMES: THE IMPROBABLE CASE OF OIL AND GAS WORKERS**

**SECTION A: Personal Bio Data**

Please Read the questions and tick (**√** ) as appropriate the correct answers that varies with the questions.

1. Gender: Male ( ) Female ( )
2. Age: 21-30years ( ) 31-40years( ) 41-50years( ) 51 – above ( )
3. Marital Status: Single ( ) Married ( ) Divorced ( ) Others ( )
4. Highest Education level: WAEC/O’LEVEL ( ) OND/NCE ( ) BSC/HND ( ) M.Sc./MBA ( ) PHD ( ) others ( )
5. For how long have you been working in this organisation? Less than 1 year ( ) 1-5 years ( ) 6-10 years ( )

11-15years ( ) 16 years - above ( )

1. Staff Status: Permanent ( ) Contract ( )
2. Department: Electrical ( ) Mechanical ( ) Drilling ( ) Maintenance crew( ) Operations  **( )**

**SECTION B: Please tick as appropriate (√ )**

The option ranges from “5-Strongly Agree”,“4-Agree”, “3-Undecided”, “2-Disagree”, “1-Strongly disagree”

| S/N | ITEMS | SA | A | U | D | SD |  |
| --- | --- | --- | --- | --- | --- | --- | --- |
|  | | 5 | 4 | 3 | 2 | 1 |  |
| **PERCEIVED USEFULNESS OF TECHNOLOGY** | | | | | | | |
| 1 | My firm adopts multiple tools to predict unexpected shutdown events leading to non-productive time |  |  |  |  |  |  |
| 2 | Our firm has long made use of sensors and analytics to monitor oil fields. |  |  |  |  |  |  |
| 3 | My firm develops a digital platform for energy commodities trading to cut costs and manage risks |  |  |  |  |  |  |
| **EMPLOYEES’ SATISFACTION** | | | | | | | |
| 1 | I have achieved the desired level of personal growth |  |  |  |  |  |  |
| 2 | I have the resources i need to do good work |  |  |  |  |  |  |
| 3 | I find the work meaningful |  |  |  |  |  |  |
| **EMPLOYEES’ COMMITMENT** | | | | | | | |
| 1 | I feel a sense of guilt about the possibility of leaving |  |  |  |  |  |  |
| 2 | I would recommend the organisation as an excellent place to work |  |  |  |  |  |  |
| 3 | I am emotionally attached to this organisation |  |  |  |  |  |  |
| **ORGANISATIONAL SUPPORT** | | | | | | | |
| 1 | My firm rewards greater effort toward meeting organisational goals |  |  |  |  |  |  |
| 2 | The organisation cares about employees’ well-being |  |  |  |  |  |  |
| 3 | My organisation organizes training programmes for the employees |  |  |  |  |  |  |
| **EMPLOYEES’ PRODUCTIVITY** | | | | | | | |
| 1 | My firm looks for ways to change processes to improve productivity |  |  |  |  |  |  |
| 2 | I exceed my daily expectations when using the right machine |  |  |  |  |  |  |
| 3 | The work environment is safe to work in |  |  |  |  |  |  |

**THANKS FOR YOUR CO-OPERATION**
